# Enhanced somatic embryogenesis in *Theobroma cacao* using the homologous BABY BOOM transcription factor

**DOI:** 10.1186/s12870-015-0479-4

**Published:** 2015-05-16

**Authors:** Sergio L Florez, Rachel L Erwin, Siela N Maximova, Mark J Guiltinan, Wayne R Curtis

**Affiliations:** Department of Chemical Engineering, The Pennsylvania State University, University Park, PA 16802 USA; Department of Plant Science and Huck Institute of Life Sciences, The Pennsylvania State University, University Park, PA 16802 USA

**Keywords:** *BABY BOOM*, Somatic embryogenesis, *Theobroma cacao*, Cell reprogramming, Plant propagation, Transient gene expression

## Abstract

**Background:**

*Theobroma cacao*, the chocolate tree, is an important economic crop in East Africa, South East Asia, and South and Central America. Propagation of elite varieties has been achieved through somatic embryogenesis (SE) but low efficiencies and genotype dependence still presents a significant limitation for its propagation at commercial scales. Manipulation of transcription factors has been used to enhance the formation of SEs in several other plant species. This work describes the use of the transcription factor *Baby Boom* (*BBM*) to promote the transition of somatic cacao cells from the vegetative to embryonic state.

**Results:**

An ortholog of the *Arabidopsis thaliana* BBM gene (*AtBBM*) was characterized in *T. cacao* (*TcBBM*). TcBBM expression was observed throughout embryo development and was expressed at higher levels during SE as compared to zygotic embryogenesis (ZE). TcBBM overexpression in *A. thaliana* and *T. cacao* led to phenotypes associated with SE that did not require exogenous hormones. While transient ectopic expression of TcBBM provided only moderate enhancements in embryogenic potential, constitutive overexpression dramatically increased SE proliferation but also appeared to inhibit subsequent development.

**Conclusion:**

Our work provides validation that *TcBBM* is an ortholog to *AtBBM* and has a specific role in both somatic and zygotic embryogenesis. Furthermore, our studies revealed that TcBBM transcript levels could serve as a biomarker for embryogenesis in cacao tissue. Results from transient expression of TcBBM provide confirmation that transcription factors can be used to enhance SE without compromising plant development and avoiding GMO plant production. This strategy could compliment a hormone-based method of reprogramming somatic cells and lead to more precise manipulation of SE at the regulatory level of transcription factors. The technology would benefit the propagation of elite varieties with low regeneration potential as well as the production of transgenic plants, which similarly requires somatic cell reprogramming.

**Electronic supplementary material:**

The online version of this article (doi:10.1186/s12870-015-0479-4) contains supplementary material, which is available to authorized users.

## Background

*Theobroma cacao*, the chocolate tree, is the basis for an 83 billion dollar a year retail chocolate industry and is a critical component of numerous economies in West Africa, South East Asia, South and Central America. This industry is predicting a shortage of cocoa (fermented and dried cacao seeds) in the near future due to an increase in chocolate demand and the recent spread of devastating *cacao* pathogens [1]. As an alternative to more traditional methods of plant propagation, somatic embryogenesis (SE) is a process that reprograms somatic cells to revert to an embryonic state, and has been used to propagate a wide diversity of *cacao* genotypes [2-4]. A high degree of genotype-dependent variation in embryogenic capacity has been observed, and remains a major obstacle for scaling this technology for commercial propagation of superior *cacao* genotypes [3].

Inducible SE was first observed in 1958 in *Daucus carota* (carrot) [5], which resulted from exposure to the synthetic auxin 2,4-dichlorophenoxyacetic acid (2,4-D). After Steward’s work with carrot, many other plants such as *Gossypium hirsutum* (cotton), *Ananas comosus* (pineapple), *Glycine max (*soy), *Capsicum annum* (sweet pepper), *Coffea arabica* (coffee), and *T. cacao* among others, have been propagated through SE [2,6-11]. In most cases, plant growth regulators were responsible for initiation of this process. Empirically identifying the correct media composition and environmental conditions can be time-consuming, tedious and variable among different species and genotypes. The lack of understanding of the mechanisms that govern this dramatic reprogramming of somatic cells represents the greatest limitation to the rational improvement of this method for the propagation of many important species, and remains a critically important aspect of producing transgenic plants.

A different approach to inducing SE that overcomes the hormone-based limitations has recently been demonstrated. The over-expression of specific regulatory genes has been identified as a tool to induce SE in several plant species (*Arabidopsis thaliana*, *Brassica napus*, *Nicotiana tabacum*, *Gossypium hirsutum*, *Capsicum annum*, and *T. cacao* among others [9,12-17]. Numerous proteins such as LEAFY COTYLEDON 1 (LEC1), LEAFY COTYLEDON 2 (LEC2), LEAFY COTYDELDON 1 LIKE (L1L), WUSCHEL (WUS), PLANT GROWTH ACTIVATOR 37 (PGA 37) and AINTEGUMENTA-LIKE 5 (AIL5) have all been shown to induce SE when overexpressed [12,18-21]. Other proteins such as AGAMOUS LIKE 15 (AGL15) and SOMATIC EMBRYOGENESIS RECEPTOR KINASE 1 (SERK1) have been shown to enhance the process of SE, resulting in an increase in the number of embryos produced [22,23].

A gene of particular interest for the manipulation of SE at the genetic level is *BABY BOOM* (*BBM*). In this work, we identify and characterize a *Theobroma cacao* gene encoding a protein with high similarity to *Arabidopsis* BBM and show its ability to induce SE. The constitutive overexpression of TcBBM resulted in a dramatic serial proliferation of somatic embryos. Furthermore, genotypes that are SE-responsive (SCA6) and non-responsive (ICS1) were studied to determine if this difference in permissiveness correlated with BBM expression patterns. This work is presented in the context of the eventual goal of systematic manipulation of the SE developmental program to improve efficiency and overcome recalcitrance for commercial plant propagation and plant improvement programs.

## Results

### Identification of BBM *T. cacao* homolog

**Figure 1:**
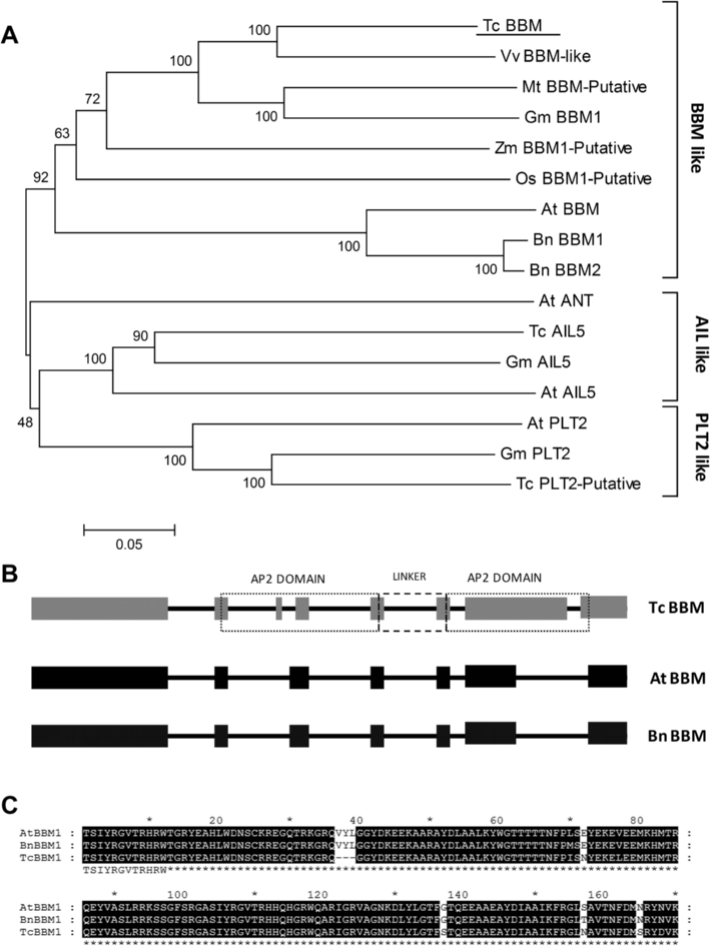
Phylogenetic analysis and gene structure of *TcBBM*. **A**. Phylogenetic analysis of AP2 gene family. The neighbor-joining consensus tree was constructed based on the full-length amino acid sequences of AP2 gene family [13,33]. The scale bar represents 0.1 substitutions per site and the values next to the nodes are the bootstrap values from 2000 replicates. **B**. Gene models of BBM genes of *Theobroma cacao* (Tc), *Arabidopsis thaliana* (At) and *Brassica napus* (Bn) are depicted by their exons (blocks) and introns (lines). The exons highlighted by the dotted lines represent the two AP2 domains, connected by the linker highlighted by the dashed lines. **C**. Alignment of the two AP2 domain repeats connected by a linker characteristic of AP2-ERF BBM genes from *Theobroma cacao* (Tc), *Arabidopsis thaliana* (At) and *Brassica napus* (Bn). At = *Arabidopsis thaliana*, Bn = *Brassica napus,* Gm = *Glycine max*, Mt = *Medicago truncatula*, Os = *Oryza sativa,* Vv = *Vitis vinifera*, Zm = *Zea mays.* BBM = BABY BOOM, AIL = AINTEGUMENTA-LIKE, ANT = AINTEGUMENTA, PLT2 = PLETHORA.

To identify a candidate for a *T. cacao* BBM homologue, a tBlastN analysis was performed against the *T. cacao* genome [24] using the *Arabidopsis* BBM (AT5G17430) protein sequence [13] as a query. The most likely candidates were then used for a phylogenetic study. As a reference, other AP2 domain genes from *T. cacao* and other species were included. Phylogenetic analysis showed candidate Tc05_t019690 (termed *TcBBM*) to be evolutionarily grouped within all the other BBM orthologs (Figure 1A). Surprisingly, *TcBBM* grouped closer to *Vitis vinifera* (grape) than to other, more evolutionarily related members of the Rosids clade (*Arabidopsis thaliana, Brassica napus* and *Medicago truncatula*). A conserved domain analysis on the amino acid sequence of TcBBM using NCBI conserved domain database [25] revealed two AP2 domains, characteristic of the AP2/ERF family of proteins that includes BABY BOOM [13]. The predicted protein sequence of TcBBM is larger (570 amino acids) than the *Arabidopsis* (AtBBM) and *Brassica napus* (BnBBM) (484 and 479 respectively) with an extra 8^th^ exon (Figure 1B). While the sequence identity of the whole coding region is only 42% with both Brassica BBMs, the two AP2 domains and their linker of *TcBBM* shared 96% amino acid identity with the *AtBBM* and *BnBBM* counterparts (Figure 1C, Additional file 1).

#### TcBBM is expressed throughout embryo development

**Figure 2:**
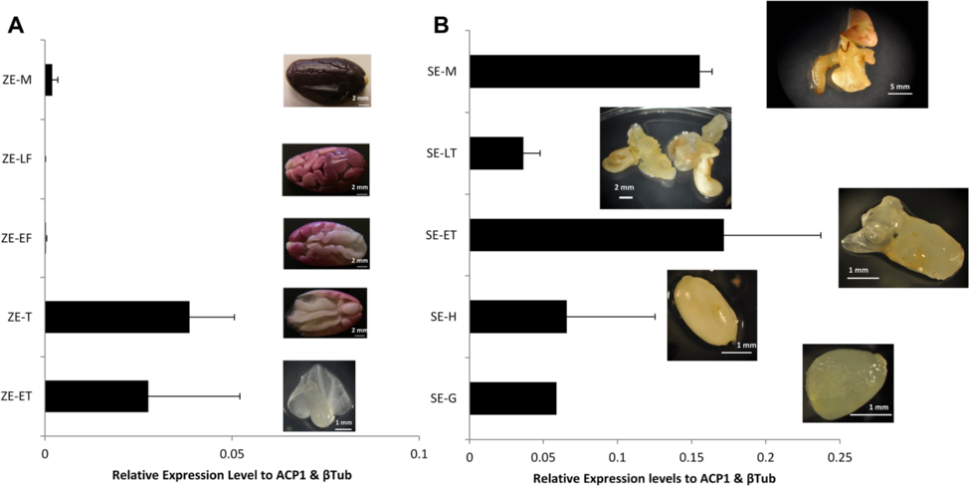
TcBBM expression throughout embryo development. Relative transcript expression of *TcBBM* throughout different development stages **A**. Zygotic embryogenesis and **B**. Somatic embryogenesis. Expression levels were analyzed by RT-qPCR and the *TcBBM* gene normalized relative to that of TcACP1 and TcβTub genes. G = globular, H = Heart, ET = Early Torpedo, LT = Late torpedo, EF = Early Full, LF = Late Full. Images for ZE-M, ZE-LF, ZE-EF and ZE-T were adapted from Maximova et al. [26].

To evaluate BBM’s expression during embryogenesis in *T. cacao*, we studied the transcript expression profiles throughout both zygotic and somatic embryo development, noting that expression is negligible in other tissue such as leaves, roots and flowers (data not shown). During zygotic embryo (ZE) development, expression was measured from five developmental time points: early torpedo (ET-ZE), late torpedo (LT-ZE), early-full (EF-ZE), late-full (LF-ZE) and mature (M-ZE) embryos (Figure 2A) as previously described [26]. For SE, globular (G-SE), heart (H-SE), early torpedo (ET-SE), late torpedo (LT-SE) and mature (M-SE) embryos were evaluated for TcBBM expression (Figure 2B). While SE and ZE were characterized by elevated expression during earlier stages, expression of TcBBM was essentially absent in the zygotic embryos after the torpedo stage, while somatic embryos displayed TcBBM expression through development until the “mature” stage (Figure 2). These results confirm the presence of TcBBM transcripts during embryogenesis in *T. cacao* and show particular importance during SE where the expression level of TcBBM was higher by almost an order of magnitude throughout SE compared to its corresponding zygotic stage; a difference that was confirmed based on an aggregate of the SE and ZE data to be statistically significant (CI >0.95).

### TcBBM is highly expressed in tissue undergoing SE

**Figure 3:**
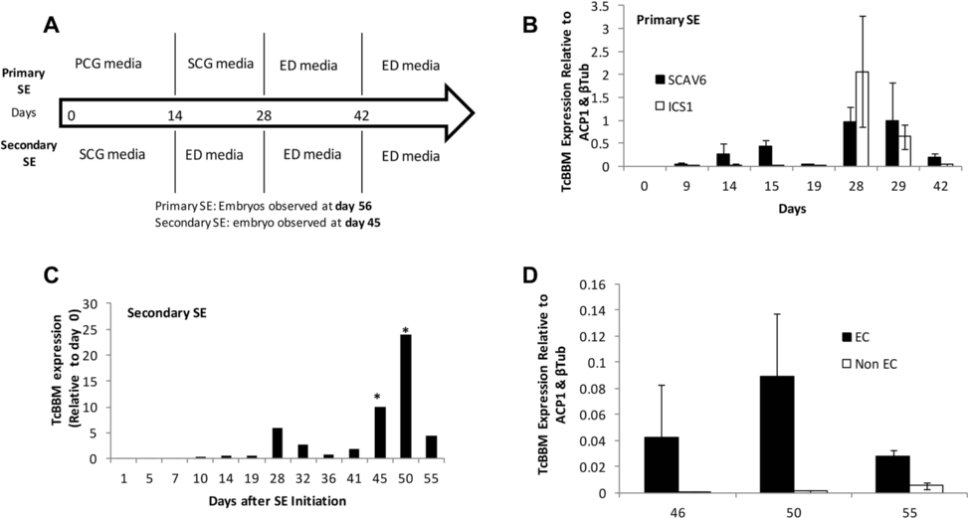
TcBBM expression throughout the process of primary and secondary embryogenesis. **A**. Schematic of the process of either primary (top) or secondary somatic (bottom) embryogenesis. PCG = Primary Callus Growth media, SCG = Secondary Callus Growth media, ED = Embryo Development media. **B**. TcBBM expression throughout primary somatic embryogenesis. **C**. TcBBM expression throughout secondary somatic embryogenesis (* represents a p-value < 0.05 for the Student’s t-test). **D**. TcBBM expression in embryonic (EC) and non-embryonic calli (Non-EC) obtained from secondary SE calli. Non-embryonic calli were classified as undifferentiated calli tissue that had not produced visible embryos up to the date the tissue was harvested. Embryogenic calli is also undifferentiated tissue; however, it is harvested from explants that had produced visible embryos. Expression levels for panels **B**, **C** and **D** were analyzed by RT-qPCR and the *TcBBM* gene normalized relative to that of TcACP1 and TcβTub genes.

BBM’s role as a possible biomarker for embryogenic tissue has been indicated in previous works [9,13-15]. To test whether TcBBM expression could be used as a biomarker for *cacao* SE initiation, we studied its gene expression levels throughout the process of primary and secondary somatic embryogenesis (Figure 3A) (A set of descriptive terms used to describe the cacao SE system are listed in Additional file 2). For primary SE, eight time points during the first six weeks of SE were studied between a responsive genotype (SCA6) and a recalcitrant genotype (ICS1). For both genotypes, TcBBM transcript was not detectable in petal tissue used to initiate primary SE. Interestingly, after culture on hormone-containing induction media, TcBBM expression was observed in SCA6 at day 9 after culture initiation (ACI), which was five days earlier than in the recalcitrant ICS1 tissue where low levels of TcBBM were detected at day 14 ACI. Throughout the first two weeks, TcBBM expression was higher in the responsive SCA6 genotype until expression in both genotypes reached comparable levels by day 28 (Figure 3B).

Secondary somatic embryos formed by hormone treatment and dedifferentiation of tissue from cotyledons of primary SEs have been shown to be more responsive and to produce a higher number of embryos than original floral somatic tissue used for initiation of primary SE [3]. To examine TcBBM’s role in these differences, TcBBM expression during secondary SE was investigated using a similar time course experiment using the responsive SCA6 genotype (Figure 3C). Expression of TcBBM was detected but did not vary significantly throughout secondary SE until a sharp increase starting after day 41 during the third transfer to embryo development (ED) media, which corresponds to the time when globular embryos were observed. Consistent with BBM expression in somatic tissue that is actively undergoing somatic reprogramming, TcBBM expression was dramatically higher in undifferentiated calli that was directly associated with tissue that had produced embryos (embryonic calli) as compared to non-embryonic calli (calli that had yet to produce any embryos when the tissue was harvested) (Figure 3D).

### TcBBM overexpression in *Arabidopsis* leads to abnormal development and an enhances somatic embryo formation

To test TcBBM functionality, the floral dip transformation method [27] was used to introduce TcBBM gene under the control of an enhanced 35S promoter (E12-Ω-CaMV-35S) [17] into *Arabidopsis thaliana* Col-0. Thirty-one E12-Ω-CaMV-35S::TcBBM transformants were confirmed by growth on selection and subsequent PCR genotyping. Since the TcBBM genomic sequence was used, RNA was extracted from these *Arabidopsis* lines to confirm proper mRNA processing. When the cDNA for *TcBBM* was sequenced, it revealed 21 fewer amino acids in the first exon compared to the predicted sequence in the *cacao* genome database (Additional file 3). This slightly-shorter-than-predicted transcript was subsequently confirmed as the native mature mRNA by analyzing the native *cacao* cDNA.

**Figure 4:**
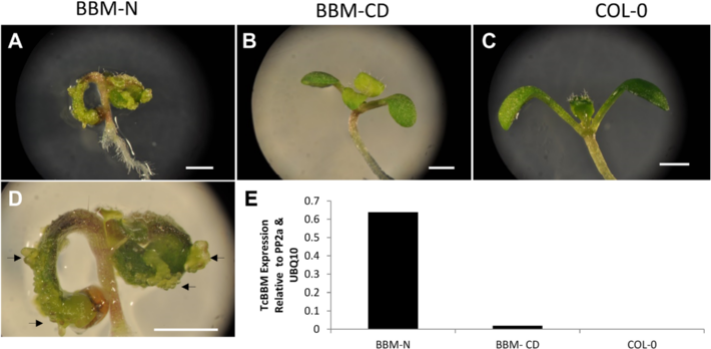
*Arabidopsis* overexpressing TcBBM leads to spontaneous regeneration from the cotyledon **A**, **D**. E12-Ω-CaMV-35S::*TcBBM (BBM-N) Arabidopsis* line showing spontaneous regeneration of cotyledon like structures from the seedling cotyledons (black arrows). **B**. E12-Ω-CaMV-35S::*TcBBM* (BBM-CD) *Arabidopsis* line showing no phenotype. **C**. *Arabidopsis* Col 0 wild type. **E**. The corresponding TcBBM levels of the three E12-Ω-CaMV-35S::*TcBBM* lines shown in images **A**, **B** and **C**. Expression levels were analyzed by RT-qPCR and the *TcBBM* gene normalized relative to *AtPP2a* and *AtUBQ10*. Image scale bars = 1 mm.

The resulting E12-Ω-CaMV-35S::TcBBM *Arabidopsis* lines exhibited a variety of phenotypes including abnormal development of leaves and cotyledons, low or no fertility, and stunted growth ranging from moderate to severe (Additional file 4). Notably, in some plants, cotyledon-like structures regenerated from the primary cotyledons (Figure 4A, D, Additional file 4). Comparable phenotypes were reported for *Arabidopsis* overexpressing the related *Brassica napus* (*BnBBM*) using a similar constitutive 35S promoter [13].

To test if there was a correlation between TcBBM expression level and the regenerative phenotype, TcBBM mRNA levels were quantified by RT-qPCR. It was observed that TcBBM expression levels were significantly higher in the plant that showed spontaneous regeneration (BBM-N) when compared to other E12-Ω-CaMV-35S::TcBBM plants that showed no phenotype (BBM-CD) (Figure 4E). Although no antibodies exist to confirm protein expression, the levels of TcBBM mRNA suggest a strong correlation between high levels of TcBBM and the formation of secondary cotyledon-like structures on *Arabidopsis* seedlings.

### Overexpression of TcBBM in *T. cacao* leads to hormone independent direct somatic embryogenesis

**Figure 5:**
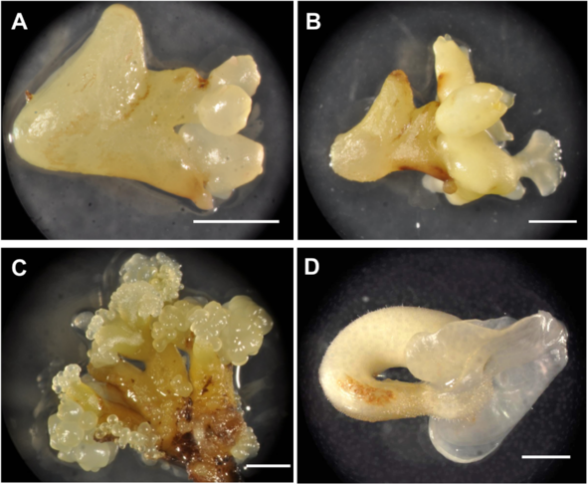
TcBBM overexpression in *cacao* leads to spontaneous direct somatic embryogenesis. **A**. 35S::TcBBM *cacao* embryo over-expressing TcBBM going through the process of spontaneous direct somatic embryogenesis. **B**. Further development of same E12-Ω-CaMV-35S::TcBBM *cacao* embryo (14 days after image on A). **C**. E12-Ω-CaMV-35S::TcBBM explant after 14 days of being subjected to hormone induced somatic embryogenesis. **D**. SCA6 wild-type *cacao* embryo showing normal cotyledon development and no spontaneous embryo regeneration. Image scale bars = 1 mm.

To observe the effects of TcBBM overexpression in *cacao*, the *TcBBM* gene was introduced under the control of the constitutive E12-Ω-CaMV-35S promoter into *cacao* cotyledons by *Agrobacterium*-mediated transformation following a published protocol utilizing hormone dependent SE initiation [28]. Since transgenic events are rare in *cacao*, a constitutive EGFP was included on the T-DNA cassette to allow for visual screening for transformants using fluorescence. Fifteen and sixteen weeks ACI, two embryos from two different explants (<0.2% of all embryos produced) showed *TcBBM* integration as detected by EGFP fluorescence and later verified via PCR based genotyping. Spontaneous SEs formed subsequently on the cotyledons of the transgenic embryos, bypassing the callus stage normally present in hormone-dependent SE (Figure 5A-B). These new embryos were characterized by abnormal cotyledon development and the serial initiation and regeneration of multiple somatic embryos (meta-embryos), the majority of which never reached normal mature SE embryo developmental stage. New meta-embryo formation was observed and was still ongoing a year after the first transgenic *TcBBM* secondary embryo was detected. A small number of TcBBM-SEs did develop “normal” cotyledons (Additional file 5) and/or an axis comparable to non-transgenic SEs. TcBBM-SEs with established axial growth (N = 4) were carefully isolated and were exposed to light and placed on conversion media (PEC) as previously described [2]. These embryos exhibited increased cotyledon growth and chlorophyll production but conversion to a new plantlet was not observed, suggesting that constitutive over-expression of TcBBM inhibits further development.

**Figure 6:**
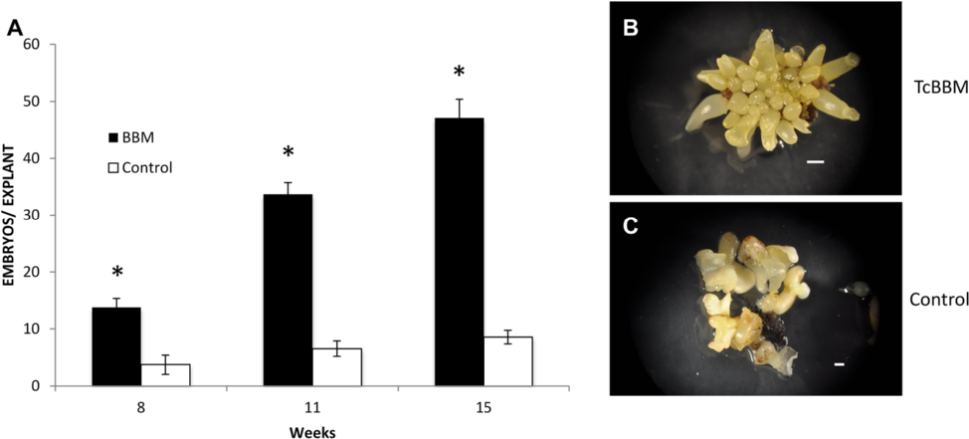
TcBBM constitutive overexpression in *cacao* leads to an increase in embryonic potential. **A**. Number of embryos produced per explant generated from E12-Ω-CaMV-35S::TcBBM or SCA6 wild-type tissue. Error bars represent one standard deviation. Image of embryos produced from **B**. E12-Ω-CaMV-35S::TcBBM or **C**. SCA6 Wt explants. Image scale bars = 1 mm. (* represents a p-value < 0.05 for the Student’s t-test).

The constitutive overexpression of TcBBM resulted in faster and increased numbers of SEs (Figure 6). When cotyledons from TcBBM-SEs were used to initiate hormone-induced SE, embryo formation was detected at 10 days ACI, reducing the time for embryo formation to almost 1/4 (Figure 3C). As the embryos continued to develop, subsequent SEs emerged directly from current embryos, something rarely seen in the wild type control. These meta-embryos most frequently developed from the embryo axis but occasionally from cotyledons (Figure 5C). To quantify this enhancement, tertiary hormone-dependent SE was initiated from isolated TcBBM-SE cotyledons. An approximate 5.5-fold increase in SEs produced per explant was observed 15 weeks ACI relative to the control regeneration from non-transgenic SE cotyledons (Figure 6A). In this experiment, the TcBBM-SE also exhibited abnormal development and did not progress towards conversion (data not shown). Unlike hormone independent SE, in this experiment, the majority of new TcBBM-SEs, which were induced on hormone-containing-medium, appeared to regenerate via indirect SE, which is characterized by an intermediate callus phase (Figure 6B). Despite the increase in *TcBBM*-SEs, the new meta-embryos also showed compromised subsequent development.

### Transient expression of TcBBM results in a higher rate of embryo production

**Figure 7:**
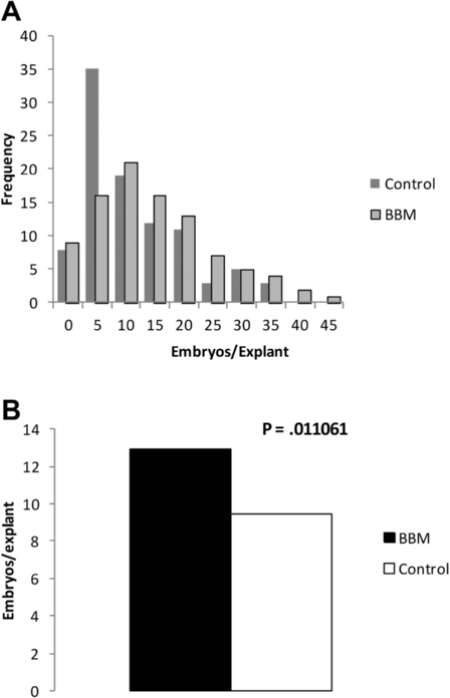
Transient expression of TcBBM in *cacao* leads to an increase of embryo produced per explant. **A**. Frequency distribution of embryos produced per explant when exposed to transient expression of *TcBBM* or control (empty vector). **B**. Average embryos/explants produced per explant in the transient TcBBM embryos and in the control. Data does not include the outliers identified by the Tukey test.

The high occurrence of abnormal development in *TcBBM*-SEs represents a limitation in using constitutive expression of this gene for plant propagation. To test a more practical approach, transient expression of TcBBM was evaluated as a strategy for improving SE. Secondary SE was initiated on SCG medium [2] from non-transgenic SE cotyledon tissue exposed to *Agrobacterium* harboring the *TcBBM* construct. Constitutively expressed *EGFP* gene was included in the construct as visual reporter of transformation efficiency. Based on the observed variable EGFP fluorescence at 1 week ACI, we deduced that the transient expression of the TcBBM was also highly variable. By week 2 ACI all the transient EGFP fluorescence was lost. Non-transgenic embryo production was counted for each explant (N = 99) throughout the 15 weeks ACI and the cumulative numbers of SEs produced by individual explants were recorded. A high degree of variability, not uncommon for SE in *cacao,* was observed. Nonetheless, a shift towards a higher number of embryos/explant occurred in the distribution for TcBBM exposed tissues (Figure 7A), resulting in an overall increase in embryo production. The tissues exposed to transient TcBBM expression had on average, 29% more SEs per explant than the control tissue, representing a total of 285 more SEs compared to the control regeneration (Figure 7B). This shift in distribution was statistically confirmed with the Kolmogorov-Smirnov (KS) test (p = 0.015) after outliers determined by Tukey’s outlier filter were removed (Additional file 6). Significantly, the resulting SEs were non-transgenic and could be converted into plantlets, indicating potential to increase embryo production efficiency in commercial scale.

## Discussion

In this work, the *BBM* homologue in *cacao* was identified through bioinformatics and subsequent functional characterization when expressed in *Arabidopsis* and *cacao*. The goal of this work was to increase our understanding of the mechanisms controlling SE in *cacao* and to explore the feasibility of using transcription factors to improve the efficiency of the somatic embryogenesis process - specifically to demonstrate enhanced non-GMO SE based transient expression.

### TcBBM ability to induce SE could be limited by its molecular environment

Overexpression of TcBBM in developing SEs clearly demonstrated an ability to activate SE pathways (Figure 5). It is puzzling why this overexpression does not lead to embryo formation when TcBBM is expressed in other tissues. For example, TcBBM was unable to induce the process of SE when constitutively over-expressed in stably transformed SCA6 suspension cells (data not shown). It would appear that TcBBM’s ability to promote SE is dependent on the physiological environment and the presence of other factors in embryogenic tissue.

While interactions among other regulators of embryogenesis have been reported, Wang’s work showing BBM as a downstream target of *FUSCA3* (*FUS3*), a B3 domain gene critical for SE and involved in embryo maturation, is the only connection between BBM and a known embryo-specific pathway [29]. Despite minimal association with other genetic components of the embryogenic pathway, overexpression of BBM has been shown to induce SE in several plant species. When the *Arabidopsis* (*AtBBM*) or *Brassica napus* (*BnBBM*) *BABY BOOM* genes were individually overexpressed in *Arabidopsis*, somatic embryos regenerated without hormone application [13]. Heterologous expression of BnBBM also successfully induced SE in *N. tabacum*, although the media required supplementation with cytokinin to achieve regeneration [14]. As an example of applying this technology, Deng et al. developed a method to overexpress the native BBM in poplar to induce SE and facilitate its propagation [15]. The tightly controlled hormone inducible promoter system based on the glucocorticoid receptor [30] was recently used with *BnBBM* to induce SE in the recalcitrant species, sweet pepper, which resulted in an increase in the number of transgenic plants produced [9]. Passarinho et al. combined a transcriptomics approach with a similar inducible BnBBM system in *Arabidopsis* to elucidate other participating genes in the SE process. Interestingly, they reported *ACTIN DEPOLYMERIZING FACTOR 9* (*ADF9*) as one of the direct targets of BBM, suggesting a link between embryo genetic reprogramming and actin-mediated cell restructuring [31]. Unfortunately, the generality of this target does not provide a specific mechanistic relationship between BBM and a SE pathway. Thus, BBM’s precise role in this extensive physiological change remains enigmatic.

Recently, Nic-Can et al., reported epigenetics, in particular methylation of histones, as a critical factor for SE [32]. Of relevance to this work, they describe a correlation between methylation patterns and expression of levels of LEC1, Wuschel-related homeobox4 (WOX4) and BBM in coffee. Expression data from a recent whole genome microarray studying transcripts levels in *cacao* leaves, roots, flowers and seed tissue also suggested possible elevated DNA methylation throughout embryogenesis [26]. The analysis indicated that a group of SET domain genes (N = 35) annotated as methyl transferases revealed similar expression levels in leaves, roots and flowers while their expression level was up-regulated in the seed, with 88% being expressed higher in seed than in any other tissue. A similar trend was observed for developing zygotic and somatic embryos where expression was higher for these methylation genes compared to levels in the leaves, roots, or flower (unpublished data). This level of regulation could help explain the tissue-dependent limitations of TcBBM. Comparing the methylation patterns of SCA6 and ICS1 in the future could provide a new insight into why certain cacao genotypes are more responsive to SE.

### *TcBBM* as a biomarker for somatic embryogenesis

TcBBM expression patterns were studied throughout primary and secondary SE as well as throughout normal zygotic embryo development. During primary SE, expression was observed earlier in the more responsive genotype, SCA6. This difference in expression could contribute to the lower embryogenic potential of ICS1 genotype as compared to SCA6. The delayed but dramatic increase in *TcBBM* gene expression in ICS1 tissue at 42 days ACI (after culture initiation) was unexpected. The reduced number of SEs produced from ICS1 genotype, suggests that TcBBM expression alone is not a sufficient indicator of the successful reprogramming of somatic cells for embryo initiation. A clear role for TcBBM in the embryogenic process is none-the-less evident based on high expression throughout embryo development as well as in the embryogenic calli but not in the non-embryogenic calli. This makes *TcBBM* expression a useful molecular biomarker for determining embryogenic tissue in *cacao* at a very early stage during SE. Additionally, *TcBBM* expression could also give a false positive indication for embryo initiation, as was the case for the ICS1 genotype. A more reliable correlation between cell reprogramming and TcBBM transcript levels might require TcBBM detection during specific times or threshold ranges, or more likely used in conjunction with additional regulator gene networks.

### TcBBM as a tool for propagation of recalcitrant genotypes

While SE represents an excellent method for propagating plants, the development of specific media and hormone requirements for each species or genotype can prove costly and time consuming. A molecular genetic manipulation approach could provide a powerful alternative for SE propagation. In this work, TcBBM has shown promise as a tool for enhancement of SE efficiency in *cacao*, in particular when expressed transiently. This strategy could also be used with other genes of similar function, in particular the *LEC2* gene, which in compliment to this work has shown analogous SE inducing ability in cacao [17]. However, transformation efficiencies in different cacao tissue still represent a large limitation to implementing this technique in recalcitrant genotypes. Petals and staminodes, which are the starting material for primary SE of cacao, displayed low transformation efficiencies. As a result, using a transient expression approach for recalcitrant genotypes remains an obstacle that will have to be developed side by side with improved DNA delivery methods. As this technology continues to be developed, there is a need for better understanding of the broader picture of embryogenic transcription factors and how they can be effectively utilized for technological purposes. For example, *LEC2*’s ability to induce SE results in a different somatic embryo phenotype and represents another interesting model to further study SE initiation. Understanding how these and other transcription factors achieve a similar feat could help understand how factors such as timing, expression levels, involvement of cofactors and chromatin remodeling control SE. Manipulation of these variables could then be used to develop a more effective strategy that can be used successfully to propagate not only *cacao* but also other crops or endangered species without generating GMO varieties.

## Conclusions

In this work, the *BABYBOOM* gene ortholog from *cacao* (*TcBBM*) was identified and functionally characterized. Expression profiling of *TcBBM* demonstrated that transcription of *TcBBM* is detected throughout both somatic and zygotic embryo development. TcBBM is highly expressed in tissue undergoing the process of SE; thus, TcBBM can be used as an embryogenesis biomarker in *cacao*. When overexpressed in both *Arabidopsis* and *cacao*, TcBBM induces embryo formation. TcBBM also displayed potential for enhancing SE via a transient expression technology. The abnormal/inhibitory phenotype of transgenic constitutive *TcBBM* provides a convenient means of excluding unwanted transgenic events when ectopic expression is being used to enhance SE. This functionally terminal phenotype increases the utility of *TcBBM* as a transient means to reprogram cells for regeneration of propagated plants that are not transgenic (non-GMO). This may also facilitate use by co-transfection and integration of only a partnered gene. Given the complexity of SE as a biological process, it is amazing that differential expression of a single gene such as *BBM* can quantitatively alter somatic embryo formation. However, BBM does not appear to be a “magic bullet” for high frequency plant propagation, and a better understanding of the complex interaction of gene regulation is needed to more effectively accomplish that goal.

## Methods

### Tissue culture for studying developmental stages of somatic embryogenesis

Somatic embryogenesis was initiated as previously described [2,3] from either petals (primary somatic embryogenesis) or cotyledons of mature somatic embryos (secondary somatic embryogenesis). For primary somatic embryogenesis, petals were taken from floral buds obtained from greenhouse grown PSU Scavina (SCA) 6–1 and ICS1 *cacao* genotypes [2]. A minimum of 15 petals was collected for each time point for each of the three replicates. Secondary somatic embryogenesis was initiated from young glossy cotyledons. Tissue was flash frozen with liquid nitrogen and stored at −80°C until RNA extraction was performed.

### Identification of TcBBM and phylogenetic tree analysis

A candidate *cacao BBM* gene was identified by searching the *cacao* genome [24] by tBLAStn using *AtBBM* (AT5G17430) as a query (E-value cut off 1e^−10^) and the top hit was selected for further analysis. The phylogenetic tree was constructed based on the full-length amino acid sequences of AP2 gene family [13,33]. The sequences were aligned using the MUSCLE software [34] and the phylogenetic tree was constructed with MEGA 4.1 [35] using the neighbor-joining algorithm with the Poisson correction distance and the pairwise deletion. The bootstrap values represent 2000 replicates.

### Cloning of *TcBBM*

Genomic DNA from SCA6 was isolated as previously described [36]. Primers TcBBM-S (5′- CGATCTAGA*ATGGCTTCCATGAACAACTGGT*-3′) and TcBBM-AS (5′-GACTCTAGACTG*TTATGTATCATTCCATACTGTGAA***-**3′) were used to amplify the *TcBBM* gene and add XbaI flanking sites. The PCR product was then blunt end ligated into the intermediate vector pSCB (Agilent Technologies, Cat 240207) as specified by the manufacturer and sequenced. The E12-Ω-CaMV-35S::-EGFP-35S terminator cassette [36] was cloned into the pCambia 1300 (Cambia Labs) vector at the HindIII and EcoRI sites creating the intermediate vector pCambia-EGFP. The EGFP coding sequence was later excised by a XbaI digestion and replaced by the *TcBBM* sequence generating the vector E12-Ω-CaMV-35S:TcBBM-pCambia to transform *Arabidopsis*. For *cacao* transformations, primers (5′-TCTAGA*ATGGCTTCCATGAACAAC*-3′ and 5′ GTTAAC*TCATGTATCATTCCATACTGTG*-3′) were used to amplify the *TcBBM* sequence and cloned into the SpeI and HpaI sites in the pGH.0126-TT2 vector (GenBank: KF871320.1). Both constructs were subsequently electroporated into *Agrobacterium tumefaciens* strain AGL1.

### *Arabidopsis Agrobacterium*–mediated transformation

Following a 2–4 day 4°C cold treatment to break dormancy, *Arabidopsis* Col-0 seeds were germinated and grown in a Conviron growth chamber (Model No. MTPS144) at 22°C with a photoperiod of 16 hours light at 200 μM/ s^2^/8 hours dark. The floral dip method was used to transform *Arabidopsis* as previously described [37]. The seeds from the resulting transformations were harvested to obtain individual transformation events. Seeds were then sterilized with a 10% bleach solution for ten minutes followed by five washes with sterile water and placed on MS basal salts (4.36 g/L Phytotechnology Laboratories®) solid medium in 10 cm plates containing 2.5% sucrose, 50 μg/mL hygromycin B (Phytotechnology Laboratories®) and 1% of agar. After 10–14 days on selection plates, plants with elongated roots and leaf development were transferred to soil and genotyped by PCR. Genotyping was performed using the Extract-N-Amp™ Plant kits (Sigma-Aldrich®) as specified by the manufacturers with the following modifications: 1. Tissue size was roughly 0.25 cm^2^ and 2. The resulting extract was diluted 1:10 before being used for a PCR reaction.

### *Cacao Agrobacterium*–mediated transformations

The procedure for transforming SCA6 *cacao* somatic embryo cotyledons was used as previously described [27] with minor modifications: *A. tumefaciens* AGL1 harboring the desired plasmid was grown to an OD_600_ of 1 instead of an OD_420_ of 0.6; the co-cultivation time with *A. tumefaciens* on the filter paper was 72 hours instead of 48 hours. SE formation was followed for fifteen weeks and all embryos produced were checked for GFP expression under a dissecting microscope to assess stable integration of the T-DNA region. Cotyledons from secondary SE were used for the *TcBBM* stable expression while cotyledons from primary SE were used for the transient expression experiment. For all transformations, glossy healthy cotyledons from mature embryos were selected.

### RT-qPCR

All total RNA extractions were done with Plant RNA reagent from Life Technologies (Cat. 12322–012) according to the manufacturer’s instructions. Total *cacao* RNA treated with RQ1 RNase-free DNase (Promega, Cat. M6101) post extraction, was used to synthesize cDNA using M-MLV reverse transcriptase (New England Biolabs, Inc., Ipswich, MA) as previously described [37]. For the primary SE time course experiment and for *Arabidopsis* comparisons, 0.5 micrograms of total RNA was used; all other experiments used one microgram of total RNA. qRT-PCR was performed as previously reported [17]. Briefly, SYBR® Premix Ex Taq™ (Clonetech cat. #RR420L) was used as suggested by the manufacturer but scaled down to final reaction volumes of 10 microliters. The cDNA was diluted 1:10 before being added to the reaction. All samples had three biological replicates unless otherwise stated. Each qPCR reaction had a technical duplicate and differences in threshold cycle (C_T_) number greater than 0.5 were reanalyzed. All reactions were carried out in the StepONEPLUS™ real time PCR system. For *cacao*, the *Acyl Carrier Protein* (*TcACP1 Accession # Tc01g039970)*), and a *Tubulin* gene in *cacao* (*TcTUB1: Accession # Tc06g000360*) were used as the reference genes. For *Arabidopsis* the gene *UBQ10* (Gene ID *AT4G05320*) and the *PP2A* subunit PDF2 (Gene ID *AT1G13320*) were used as the reference genes as specified by Czechowski et al. [38]. The primers used to detect TcBBM transcript were designed based on the coding sequence of *TcBBM* (Tc05_t019690). (TcBBM-F 5′-GGTGCAAGCAGGAGCAAGATTCTG3, TcBBM-R 5′GAGCTATGCTCCATTGAAGAAGAGTCC3′). TcBBM primer efficiency was calculated using the inverse of the slope of a “C_t_ vs. Signal” plot (Efficiency = 10 ^1/slope^ -1) [39]. Four serial dilutions yielding ten samples in triplicate were used and the estimated efficiencies were 77% and 80% for SCA6 and ICS1 genotype, respectively.

### Statistics

All statistical analysis were performed using the Mathworks’® Matlab (R2014a) software. The Tukey’s filter for outliers was applied to identify outliers in both the BBM and the control data sets. The Shapiro-Wilk test for normality was also performed. The Kolmogorov-Smirnov (KS) test was performed on both data sets before and after removal of the outliers, showing significant distribution differences in both cases.

### Availability of supporting data

Sequence data from this article can be found in either The *Arabidopsis* Information Resource (TAIR) or CocoaGenDB (http://cocoagendb.cirad.fr).

Data for the phylogenetic analysis (alignment and tree) can be found in TreeBASE (http://treebase.org/treebase-web/search/study/summary.html?id=17220).

## Additional files

Additional file 1:
**Full-length amino acid alignment of the** ***Theobroma cacao (Tc), Arabidopsis thaliana (At) and Brassica napus (Bn) BBM.*** Identity is shown in black while similarity is shown in gray. The dashed area represents the two AP2 DNA binding domains joined by a linker shown in dotted lines. Alignment was done by MUSCLE software [34]. Additional file 2:
**Definition of terms associated with somatic embryogenesis.**
Additional file 3:
***TcBBM*** **sequence has 21 fewer amino acids than predicted.** A. Gene model of *TcBBM*. B. Alignment of the correct coding sequence of *TcBBM* (Top sequence) and the predicted *TcBBM* (bottom) from *cacao* genome database (http://cocoagendb.cirad.fr/). The letters highlighted in grey show the 21 amino acids that were improperly predicted. Additional file 4:
**Phenotypes for the TcBBM heterologous overexpressing E12-Ω-CaMV-35S::** ***TcBBM Arabidopsis*** **lines.** TcBBM overexpression leads to a stunted growth phenotype in the transgenic lines (A) as well as in abnormal cotyledon development (B) and the spontaneous regeneration of cotyledon-like structures from seedling cotyledons (C). Image scale bar = 1 mm. Additional file 5:
**Constitutive overexpressing TcBBM embryo leads to abnormal embryo development in** ***cacao*** **.** A. Mature *TcBBM* overexpressing *cacao* SE after several weeks on conversion medium, incubated in the light. B. EGFP expression confirms continued expression from the T-DNA cassette. Additional file 6:
**Two-sample Kolmogorov–Smirnov test.** The results of the Kolmogorov-Smirnov (KS) test comparing the distribution of the TcBBM-SEs and the control SE data sets for the number of embryos regenerated per explant. The KS test reported a value of 0.2182 for the maximum difference between the cumulative distributions (D) and shows a statistical difference in distribution (p-value = 0.015) between data sets. Since there were visually a few extreme outliers at high embryo per explant values (not uncommon for SE studies), the data was examined for the nature of distribution and outliers using available statistical tests. Tukey’s test for outliers revealed five outliers for the TcBBM-SE dataset and four for the control data set. Shapiro-Wilk test for normality revealed non-normal distributions for both the TcBBM-SE and the control data set with p-values of 1.1×10^−7^ and 6.5×10^−14^.

## Abbreviations

2,4-D: 2,4-Dichlorophenoxyacetic acid
ABI3: ABA INSENSITIVE 3
ACP1: Acyl carrier protein
AGL15: AGAMOUS-like 15
Ail: AINTEGUMENTA-Like
BBM: BABY BOOM
ED: Embryo development media
FUS3: FUSCA3
LEC: Leafy cotyledon
L1L: Leafy cotyledon 1 like
PCG: Primary callus growth media
PEC: Primary embryo conversion media
PGA37: Plant growth activator 37
SCA6: Scavina 6
SCG: Secondary callus growth media
SE: Somatic embryogenesis
TUB1: Tubulin1
Wus: WUSCHEL
ZE: Zygotic embryogenesis
