# Supplementary figures and images for: Enhanced somatic embryogenesis in Theobroma cacao using the homologous BABY BOOM transcription factor
Source: BMC Plant Biol. 2015 May 16;15:121. doi: 10.1186/s12870-015-0479-4 (PMC4449528; doi:10.1186/s12870-015-0479-4)

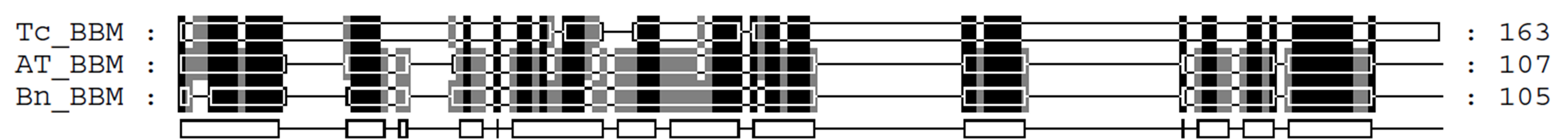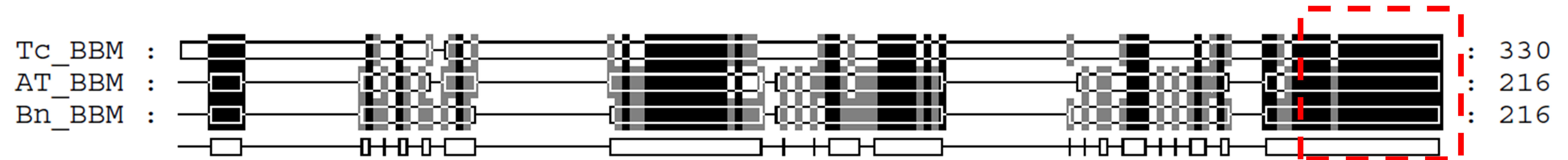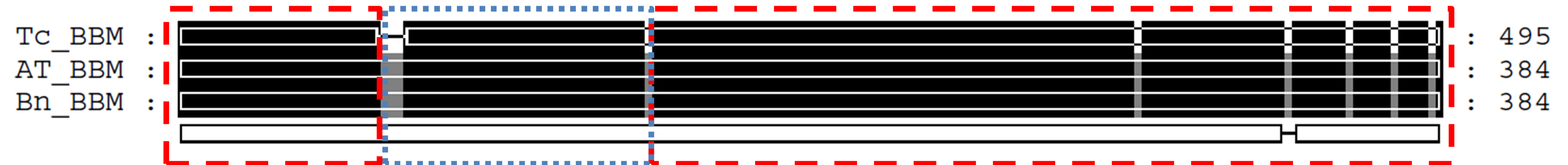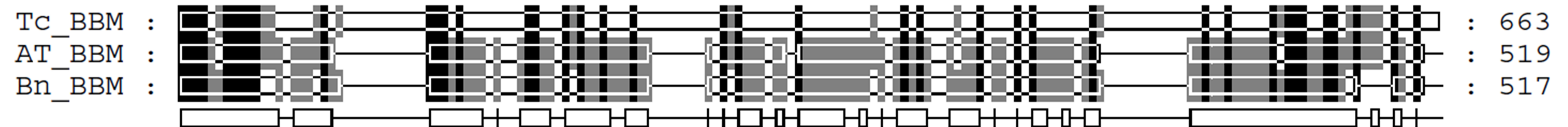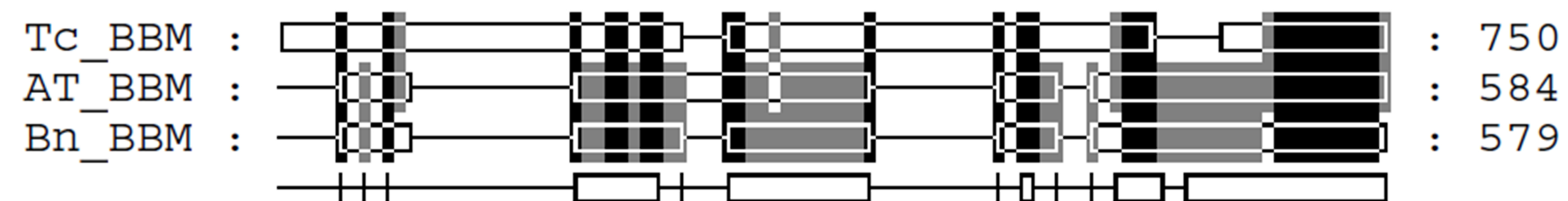

**BBM alignment with AtBBM**  
**42% identity in protein sequence**  
**96% identity in AP2 DNA binding domain**

Supplement: Additional file 1: — Full-length amino acid alignment of the Theobroma cacao (Tc), Arabidopsis thaliana (At) and Brassica napus (Bn) BBM. Identity is shown in black while similarity is shown in gray. The dashed area represents the two AP2 DNA binding domains joined by a linker shown in dotted lines. Alignment was done by MUSCLE software [34]. [file 12870_2015_479_MOESM1_ESM.pdf]

**A****B****C**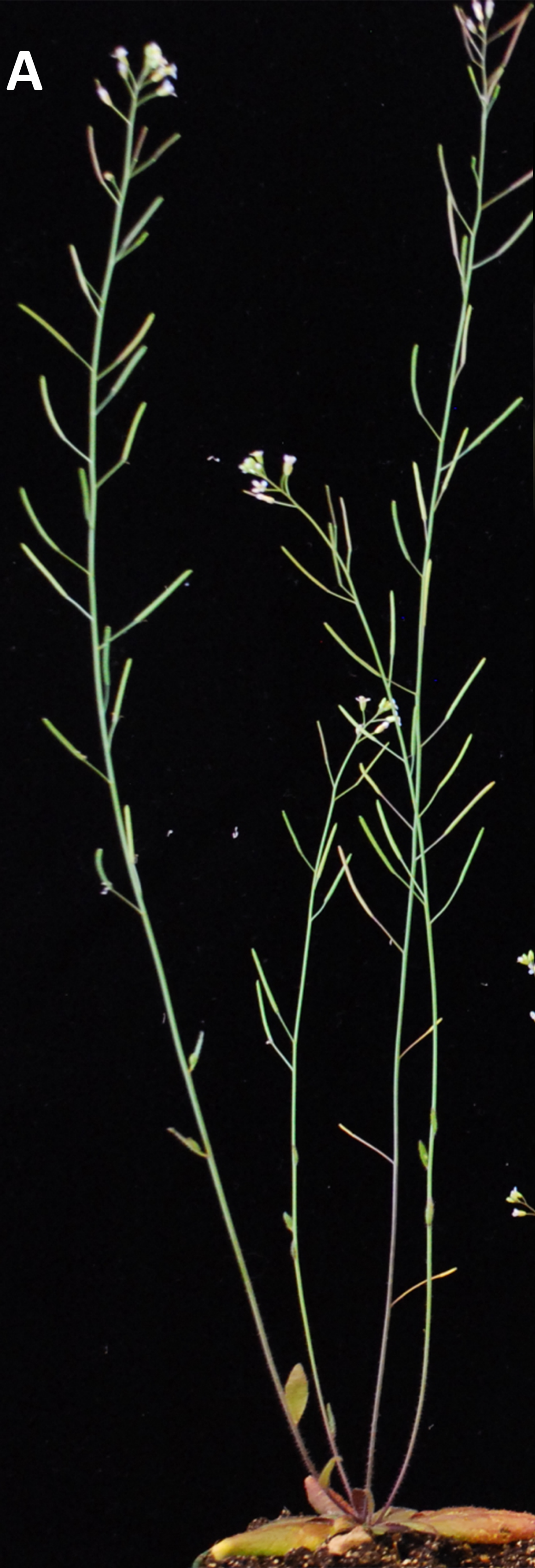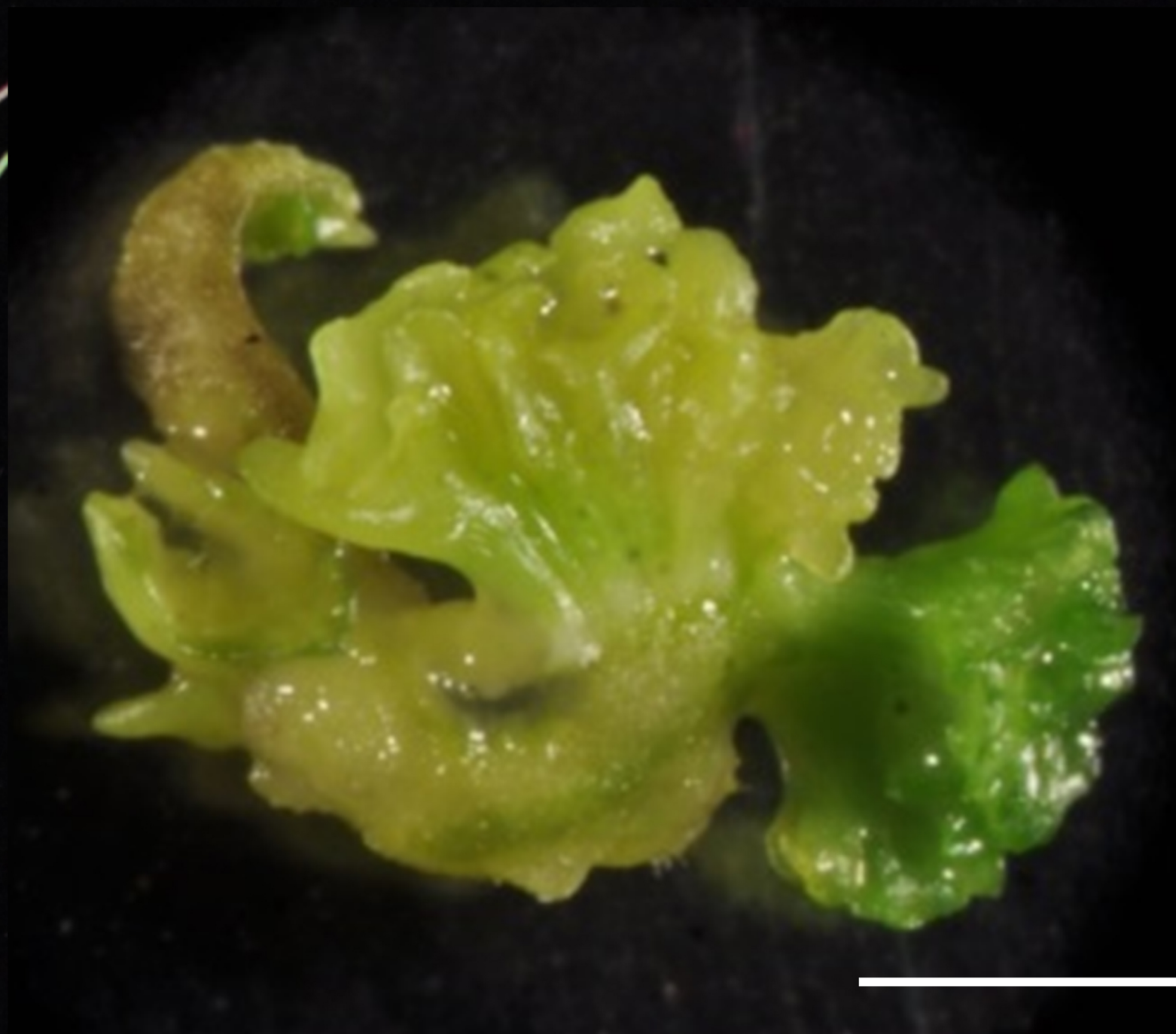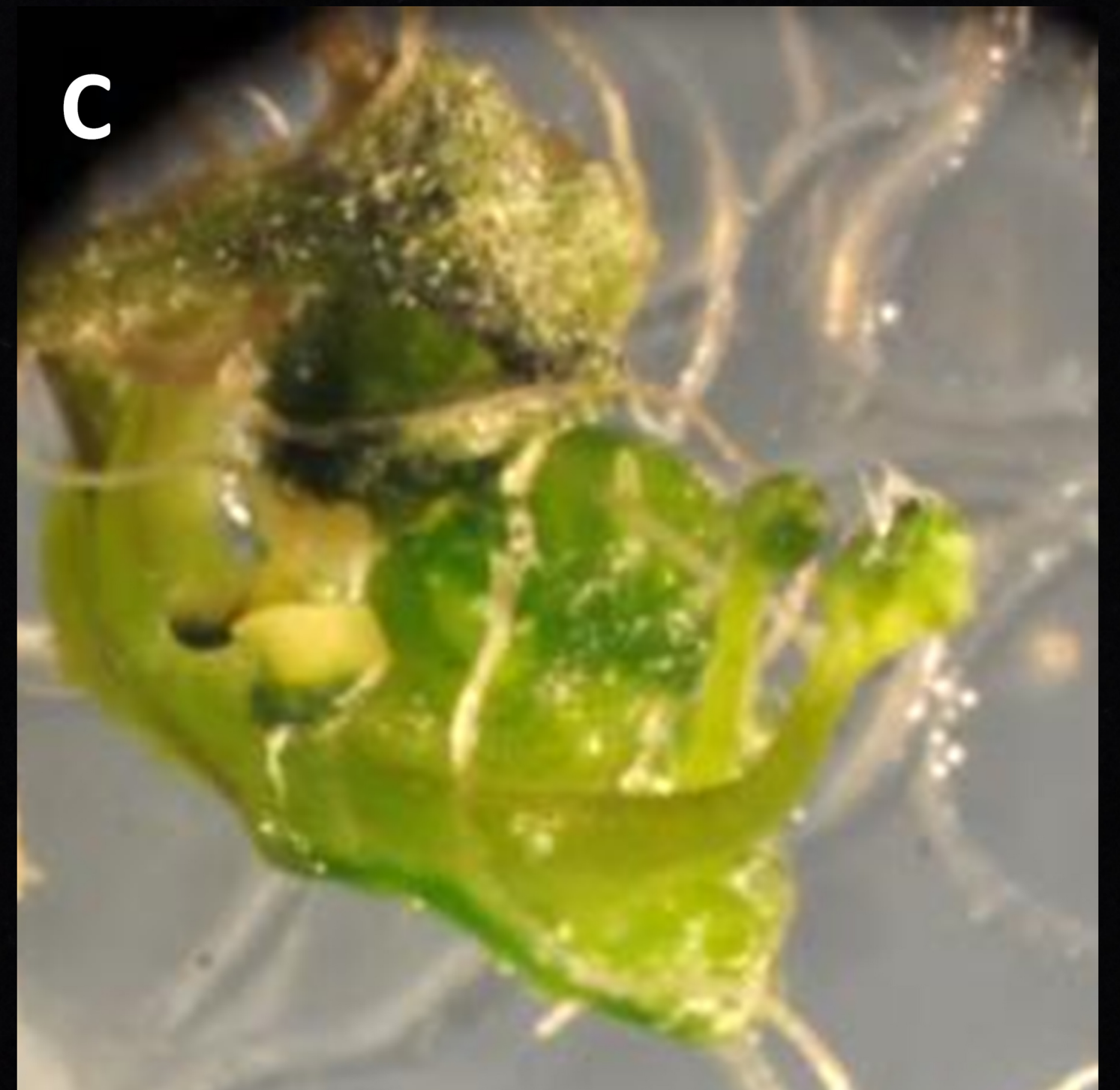**Col 0****Line CA****Line AB****Line AA****Line CB**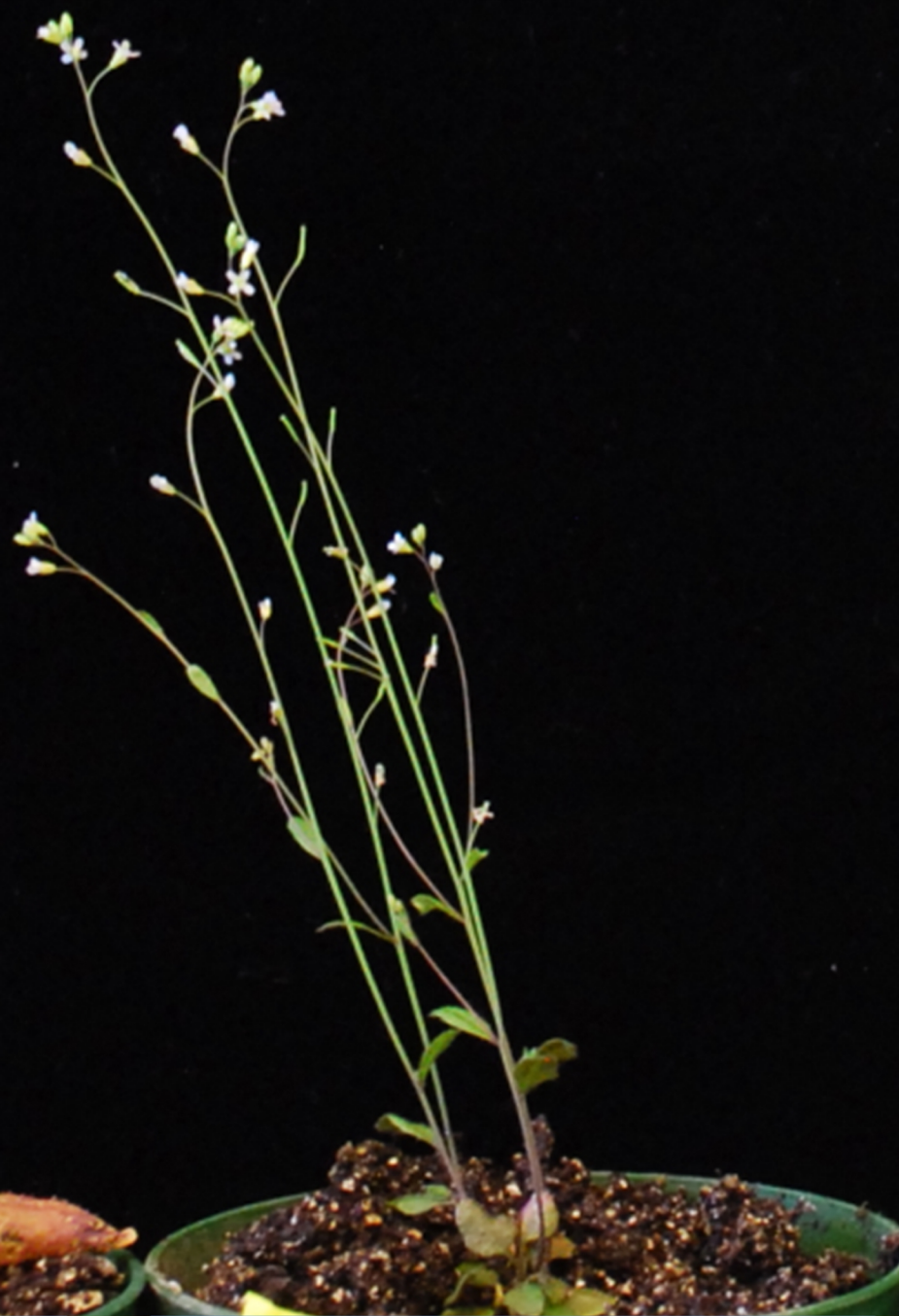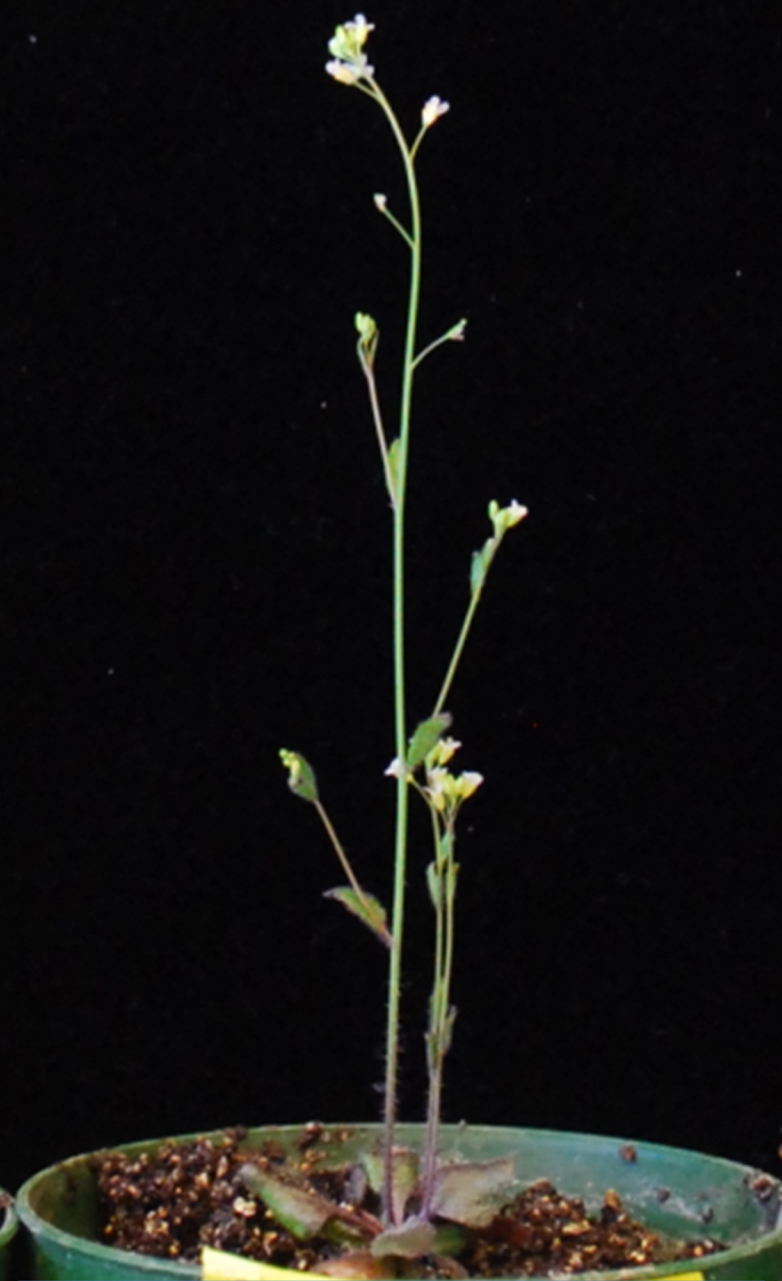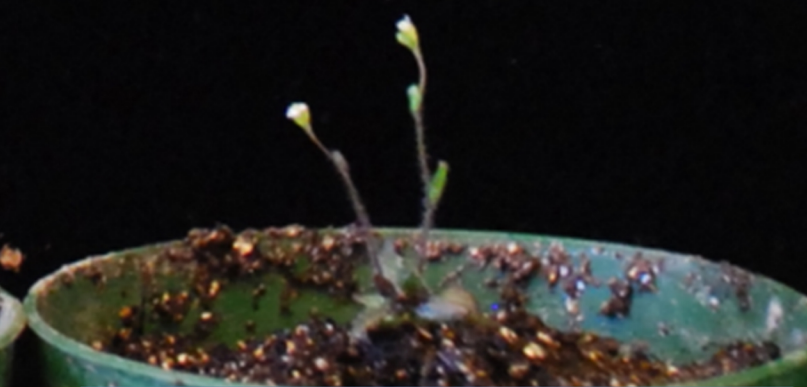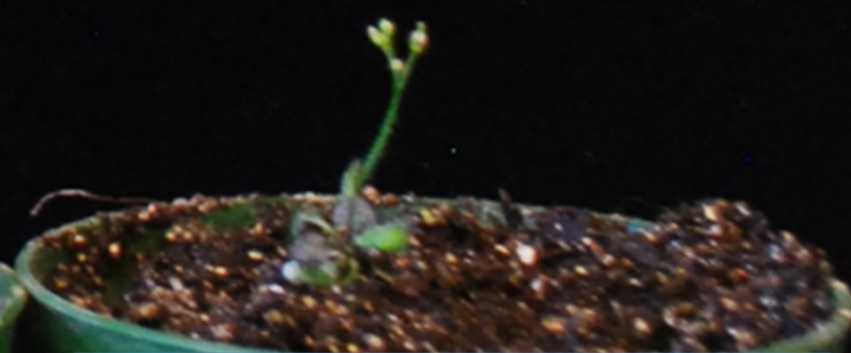

Supplement: Additional file 4: — Phenotypes for the TcBBM heterologous overexpressing E12-Ω-CaMV-35S:: TcBBM Arabidopsis lines. TcBBM overexpression leads to a stunted growth phenotype in the transgenic lines (A) as well as in abnormal cotyledon development (B) and the spontaneous regeneration of cotyledon-like structures from seedling cotyledons (C). Image scale bar = 1 mm. [file 12870_2015_479_MOESM4_ESM.pdf]

**A**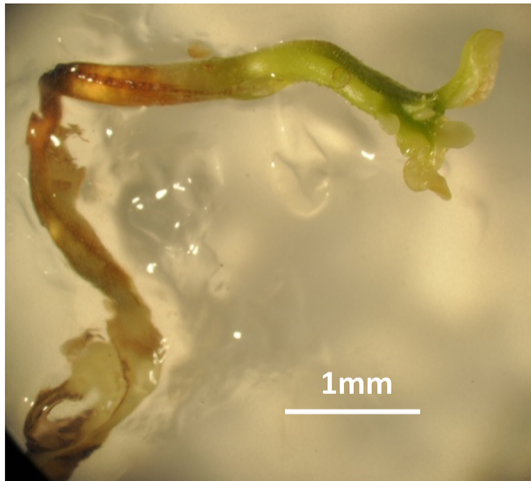**B**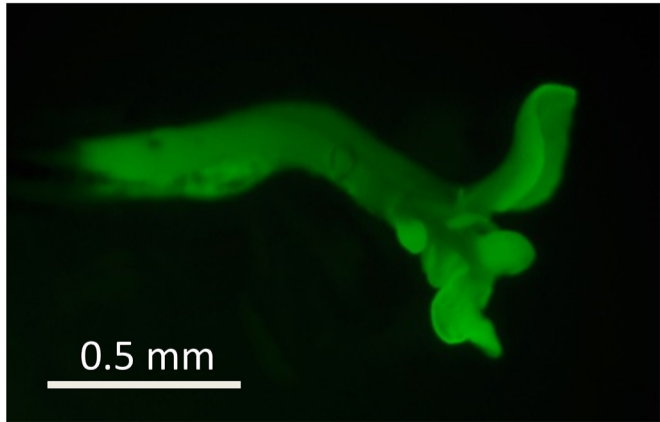

Supplement: Additional file 5: — Constitutive overexpressing TcBBM embryo leads to abnormal embryo development in cacao . A. Mature TcBBM overexpressing cacao SE after several weeks on conversion medium, incubated in the light. B. EGFP expression confirms continued expression from the T-DNA cassette. [file 12870_2015_479_MOESM5_ESM.pdf]

# Empirical CDF

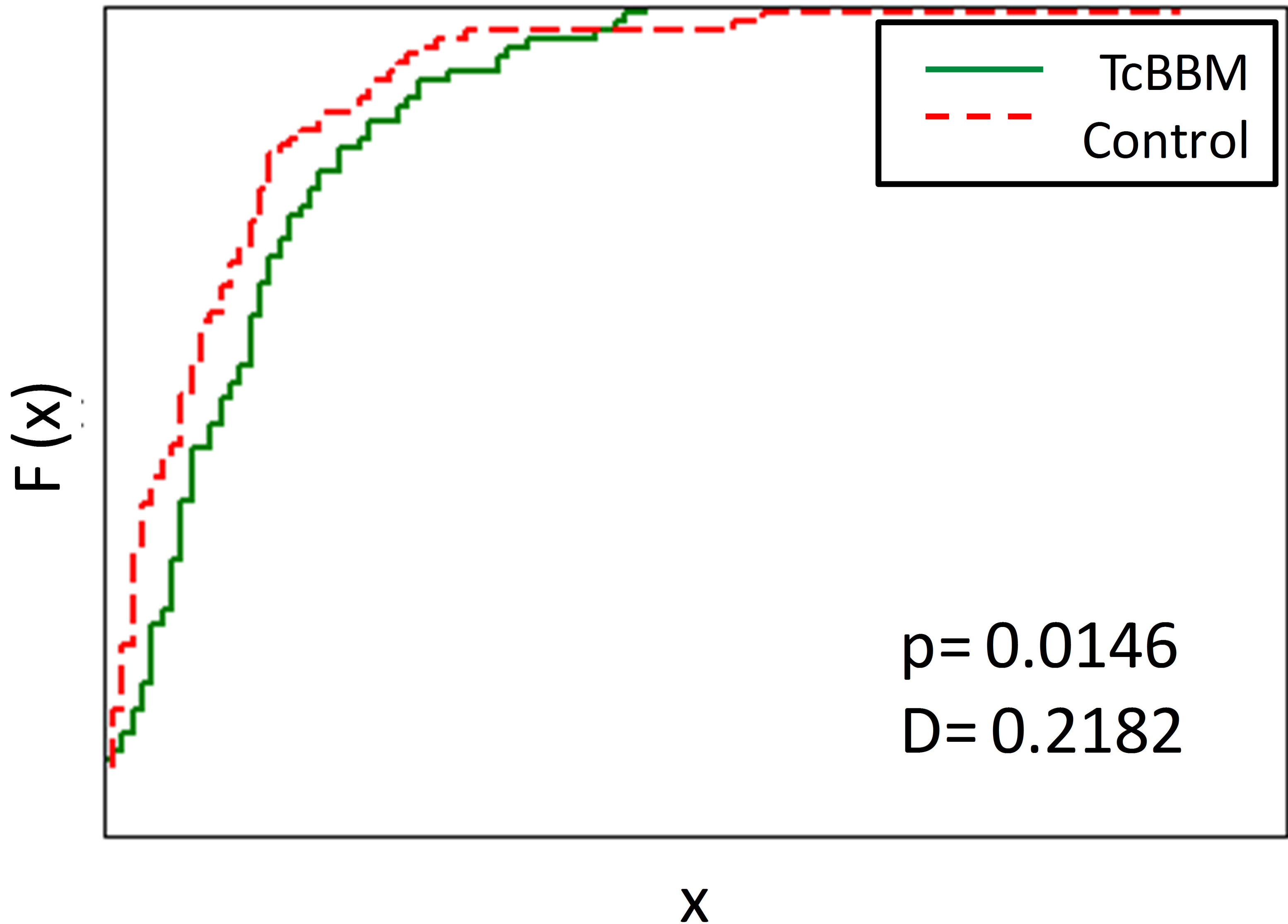

Supplement: Additional file 6: — Two-sample Kolmogorov–Smirnov test. The results of the Kolmogorov-Smirnov (KS) test comparing the distribution of the TcBBM-SEs and the control SE data sets for the number of embryos regenerated per explant. The KS test reported a value of 0.2182 for the maximum difference between the cumulative distributions (D) and shows a statistical difference in distribution (p-value = 0.015) between data sets. Since there were visually a few extreme outliers at high embryo per explant values (not uncommon for SE studies), the data was examined for the nature of distribution and outliers using available statistical tests. Tukey’s test for outliers revealed five outliers for the TcBBM-SE dataset and four for the control data set. Shapiro-Wilk test for normality revealed non-normal distributions for both the TcBBM-SE and the control data set with p-values of 1.1×10^−7^ and 6.5×10^−14^. [file 12870_2015_479_MOESM6_ESM.pdf]
