# Supplementary material for: Enhanced somatic embryogenesis in Theobroma cacao using the homologous BABY BOOM transcription factor
Source: BMC Plant Biol. 2015 May 16;15:121. doi: 10.1186/s12870-015-0479-4 (PMC4449528; doi:10.1186/s12870-015-0479-4)
Supplement: Additional file 2: — Definition of terms associated with somatic embryogenesis. [file 12870_2015_479_MOESM2_ESM.docx]

**Additional File 1. Definition of terms associated with somatic embryogenesis**

**Hormone-dependent/induced SE:** refers to any formation of somatic embryos that resulted hormone-containing media. This process is usually characterized by the reprogramming of the tissue to generate callus from which the somatic embryo is formed.

**Hormone-independent SE**: Refers to the formation of embryos without the presence of any external hormones.

**Meta-embryos:** Refers to the new embryos that have been produced from the over-expression of TcBBM. Because embryos are constantly being produced, their stage cannot be tracked and thus referred as meta-embryos.

**Primary somatic embryogenesis:** Somatic embryogenesis that uses primary tissue (petals or staminodes) to produce somatic embryos.

**Primary somatic embryo:** Embryo produced from primary somatic embryogenesis

**Secondary somatic embryogenesis**: Somatic embryogenesis that uses secondary tissue (cotyledons from primary embryos) to produce somatic embryos.

**Secondary somatic embryos**: Embryo produced from secondary somatic embryogenesis.

**Tertiary somatic embryogenesis**: Somatic embryogenesis that uses secondary tissue (cotyledons from primary embryos) to produce somatic embryos.

**Tertiary somatic embryos**: Embryo produced from secondary somatic embryogenesis.
