# Supplementary material for: Enhanced somatic embryogenesis in Theobroma cacao using the homologous BABY BOOM transcription factor
Source: BMC Plant Biol. 2015 May 16;15:121. doi: 10.1186/s12870-015-0479-4 (PMC4449528; doi:10.1186/s12870-015-0479-4)
Supplement: Additional file 3: — TcBBM sequence has 21 fewer amino acids than predicted. A. Gene model of TcBBM. B. Alignment of the correct coding sequence of TcBBM (Top sequence) and the predicted TcBBM (bottom) from cacao genome database (http://cocoagendb.cirad.fr/). The letters highlighted in grey show the 21 amino acids that were improperly predicted. [file 12870_2015_479_MOESM3_ESM.pdf]

A

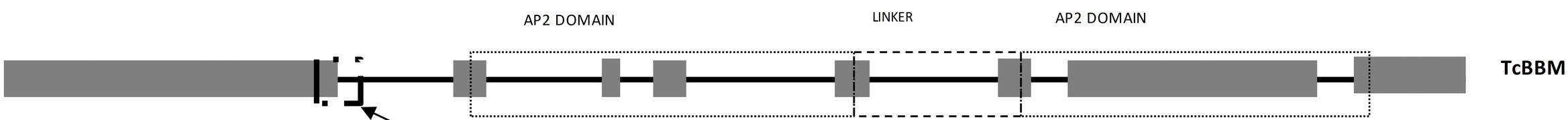

B

```
          *         20        *         40        *         60        *         80        *        100        *        120        *        140        *        160        *
: MASMNNWLAFSLSPQELPSQTVDQDHHSQTAVSRLGFNSDDISGADVSGECFDLTSDSSAPSLNLPPPFGILEAFNRNNQSQ----------DWNMKGLGMNSDGNYKTSSELSMLMGSSCNGQSLDQSNQEPKLENFLGNHSFSNHQQNKLHGCNTMYNTTTGE : 155
: MASMNNWLAFSLSPQELPSQTVDQDHHSQTAVSRLGFNSDDISGADVSGECFDLTSDSSAPSLNLPPPFGILEAFNRNNQSQSSVCFAPSKHTRTFSSFSHVKDWNMKGLGMNSDGNYKTSSELSMLMGSSCNGQSLDQSNQEPKLENFLGNHSFSNHQQNKLHGCNTMYNTTTGE : 176
  MASMNNWLAFSLSPQELPSQTVDQDHHSQTAVSRLGFNSDDISGADVSGECFDLTSDSSAPSLNLPPPFGILEAFNRNNQSQ DWNMKGLGMNSDGNYKTSSELSMLMGSSCNGQSLDQSNQEPKLENFLGNHSFSNHQQNKLHGCNTMYNTTTGE

 180        *        200        *        220        *        240        *        260        *        280        *        300        *        320        *        340        *
: YMFPNCSLQLPSEDTTNARTSNGGDDNDNNNNKNNNNNTNINTGNGSSSIGLSMIKTWLRNQPAPPQPEAKNNGGASQSLSLSMSTGSQTGSPLPLLTSSTGGGSGGESSSSDNNKQQKTPTGMDSESGAIEAMPRKSIDTFGQRTSIYRGVTRHRWTGRYEAHLWDNSCRREGQT : 331
: YMFPNCSLQLPSEDTTNARTSNGGDDNDNNNNKNNNNNTNINTGNGSSSIGLSMIKTWLRNQPAPPQPEAKNNGGASQSLSLSMSTGSQTGSPLPLLTSSTGGGSGGESSSSDNNKQQKTPTGMDSESGAIEAMPRKSIDTFGQRTSIYRGVTRHRWTGRYEAHLWDNSCRREGQT : 352
  YMFPNCSLQLPSEDTTNARTSNGGDDNDNNNNKNNNNNTNINTGNGSSSIGLSMIKTWLRNQPAPPQPEAKNNGGASQSLSLSMSTGSQTGSPLPLLTSSTGGGSGGESSSSDNNKQQKTPTGMDSESGAIEAMPRKSIDTFGQRTSIYRGVTRHRWTGRYEAHLWDNSCRREGQT

     360        *        380        *        400        *        420        *        440        *        460        *        480        *        500        *        520
: RKGRQGGYDKEEKAARAYDLAALKYWGTTTTTNFPISNYEKELEEMKHMTRQEYVASLRRKSSGFSRGASIYRGVTRHHQHGRWQARIGRVAGNKDLYLGTFSTQEEAAEAYDIAAIKFRGLNAVTNFDMSRYDVKSILESSTLPIGGAAKRLKDVEQAEMALDVQRVDDDNMSSQ : 507
: RKGRQGGYDKEEKAARAYDLAALKYWGTTTTTNFPISNYEKELEEMKHMTRQEYVASLRRKSSGFSRGASIYRGVTRHHQHGRWQARIGRVAGNKDLYLGTFSTQEEAAEAYDIAAIKFRGLNAVTNFDMSRYDVKSILESSTLPIGGAAKRLKDVEQAEMALDVQRVDDDNMSSQ : 528
  RKGRQGGYDKEEKAARAYDLAALKYWGTTTTTNFPISNYEKELEEMKHMTRQEYVASLRRKSSGFSRGASIYRGVTRHHQHGRWQARIGRVAGNKDLYLGTFSTQEEAAEAYDIAAIKFRGLNAVTNFDMSRYDVKSILESSTLPIGGAAKRLKDVEQAEMALDVQRVDDDNMSSQ

 *        540        *        560        *        580        *        600        *        620        *        640        *        660        *        680        *        700
: LTDGINNYGAAHHGWPTIAFQQAQPFSMHYPYGQRVWCKQEQDSDANHTFQDLHQLQLGSTHNFFQPSVLHNLMAMDSSSMEHSSGSNSVIYCNGGGGDAAGSNGASGSYQAVGYGGNGGYVIPMGTVVASDSNQNQGNGFGDNEVKTLGYETMYGSADPYHPRNLYYLSQQSSTG : 683
: LTDGINNYGAAHHGWPTIAFQQAQPFSMHYPYGQRVWCKQEQDSDANHTFQDLHQLQLGSTHNFFQPSVLHNLMAMDSSSMEHSSGSNSVIYCNGGGGDAAGSNGASGSYQAVGYGGNGGYVIPMGTVVASDSNQNQGNGFGDNEVKTLGYETMYGSADPYHPRNLYYLSQQSSTG : 704
  LTDGINNYGAAHHGWPTIAFQQAQPFSMHYPYGQRVWCKQEQDSDANHTFQDLHQLQLGSTHNFFQPSVLHNLMAMDSSSMEHSSGSNSVIYCNGGGGDAAGSNGASGSYQAVGYGGNGGYVIPMGTVVASDSNQNQGNGFGDNEVKTLGYETMYGSADPYHPRNLYYLSQQSSTG

     *        720        *        740        *
: GVKASSYDQASACNNWVPTAVPTIAQRSSNMAVCHGAPTFTVWND- : 728 ← Correct TcBBM cDNA sequence
: GVKASSYDQASACNNWVPTAVPTIAQRSSNMAVCHGAPTFTVWNDT : 750 ← Predicted TcBBM cDNA sequence
  GVKASSYDQASACNNWVPTAVPTIAQRSSNMAVCHGAPTFTVWND
```
